# First cytogenetic report in *Cichlasoma sanctifranciscense* Kullander, 1983 (Perciformes, Cichlidae) from northeastern Brazil with inferences on chromosomal evolution of Cichlasomatini

**DOI:** 10.3897/CompCytogen.v9i4.5562

**Published:** 2015-10-07

**Authors:** Leandro A. Argôlo, Paulo Roberto Antunes de Mello Affonso

**Affiliations:** 1 Universidade Estadual do Sudoeste da Bahia, Campus de Jequié, Laboratório de Citogenética, Avenida José Moreira Sobrinho s/n, Jequiezinho, 45.206-190 Jequié, BA, Brazil

**Keywords:** Chromosomes, Cichlasomatini, Cytotaxonomy, Ichthyofauna

## Abstract

Even though genetic aspects of some cichlids have been widely studied over the last decades, little is known about the genomic structure of Cichlidae when compared to the large number of species in the family. In this paper, the first chromosomal data for *Cichlasoma sanctifranciscense* Kullander, 1983 are presented and discussed based on cytotaxonomic and karyoevolutionary inferences on Cichlasomatini. All individuals shared a diploid number of 2n=48 distributed as 10sm+28st+10a and Ag-NORs on short arms of a submetacentric pair. Heterochromatin was detected at pericentromeric regions of most chromosomes and at terminal sites of a few pairs. GC-rich regions were observed on short arms of two biarmed pairs, including the pair bearing Ag-NORs. Double-FISH with ribosomal probes revealed 18S rDNA clusters coincident with GC-rich regions in two biarmed pairs and 5S rDNA at interstitial location of an acrocentric pair. *Cichlasoma sanctifranciscense* shares some symplesiomorphic traits described in Cichlidae (2n=48 and pericentromeric C-bands) while other chromosomal features diverge from the common trend reported in Cichlasomatini, such as multiple 18S rDNA sites combined with high FN values. Finally, the present results are useful to support taxonomic identification once species-specific markers have been provided in *Cichlasoma sanctifranciscense*.

## Introduction

Cichlids are one of the largest families within vertebrates, including more than 1600 species (Froese and Pauly 2015) and have been regarded as model organisms for evolutionary, genetic and ecological studies. In the Neotropical region, this group is represented exclusively by the monophyletic subfamily Cichlinae that stands out as the third most predominant group of freshwater fish (Reis et al. 2003).

Because of their explosive adaptive radiation (Smith et al. 2008), comparative cytogenetic studies in cichlids are particularly interesting for inferences on chromosomal evolution and cytotaxonomy. Yet, the number of karyotyped species in Cichlidae is small when compared to the remarkable diversity of this family, comprising only about 8% of described species (Feldberg et al. 2003, Valente et al. 2012). Moreover, most karyotypic reports in this fish group include only conventional chromosomal analyses, while detailed information such as mapping of specific genes or regions are restricted to a few species (e.g. Perazzo et al. 2011, Schneider et al. 2012, Schneider et al. 2013).

A compilation of the chromosomal dataset in this family revealed that more than 60% of karyotypes in Cichlidae follow the plesiomorphic condition proposed for the order Perciformes, i.e. 48 chromosomes, mostly acrocentric (Thompson 1979, Feldberg et al. 2003, Poletto et al. 2010, 2012). On the other hand, cichlids with highly divergent karyotypes have been recently reported in this family, like that observed in genus *Symphysodon* Heckel, 1840 whose species are characterized by 2n=60, several biarmed chromosomes and meiotic chains (Gross et al. 2009, 2010).

A relatively high number of cytogenetic reports is available in cichlids of the tribe Cichlasomatini (35 species). These data (see Suppl. material 1: Table S1) indicate a remarkable chromosomal variation (mainly pericentric inversions) that contrasts with the narrow ecomorphological diversity of Cichlasomatini in relation to other tribes like Geophagini and Heroini (López-Fernándes et al. 2013). Such discrepancy between genome organization and variation in external morphology reinforces the potential of cytogenetic data to assess evolutionary trends and speciation processes in this tribe.

Therefore, cytogenetic studies based on distinct banding methodologies and mapping of ribosomal genes were performed in populations of *Cichlasoma sanctifranciscense* Kullander, 1983 along isolated hydrographic basins in northeastern Brazil. Besides increasing the chromosomal data in Cichlidae, these results have proved to be informative to evolutionary and cytotaxonomic inferences in Cichlasomatini.

## Material and methods

**Figure 1.**
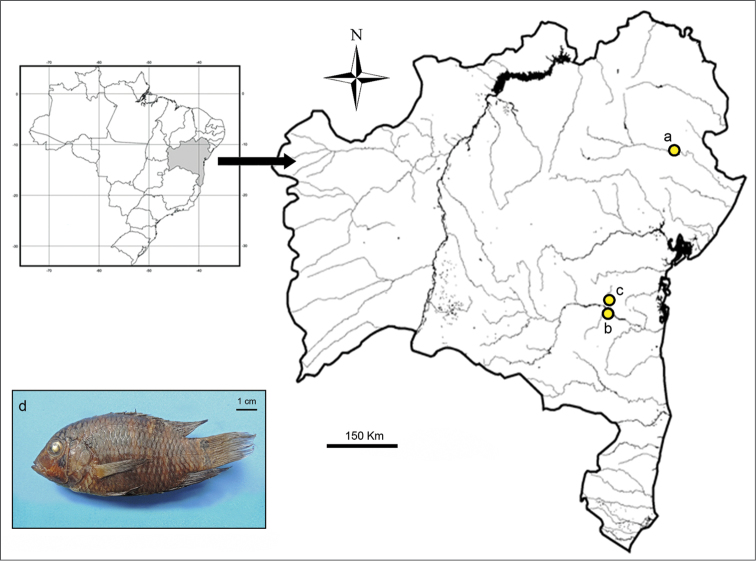
Map of state of Bahia, northeastern Brazil indicating the collection sites in Itapicuru-mirim (**a**), Contas (**b**) and Preto do Crisciúma (**c**) rivers of *Cichlasoma sanctifranciscense* specimens (**d**).

Twenty-one specimens of *Cichlasoma sanctifranciscense* were collected along three rivers from two large coastal hydrographic basins in Bahia, northeastern Brazil. The sampled rivers were: Contas River (eight males, three females and three juveniles) and Preto do Crisciúma River (two males), both within the Contas River Basin; and Itapicuru-mirim River (four females and one male) in the Itapicuru River basin (Fig. 1). Voucher specimens are deposited in the fish collection from the Zoology Museum at Universidade de Sгo Paulo (MZUSP 95173).

Direct metaphase preparations were obtained from kidney cells (Bertollo et al. 1978) after immunostimulation of collected specimens for 48–72 h (Molina et al. 2010). Prior to this procedure, all individuals were euthanized by immersion in tap water at 0–4 °C up to complete interruption of gill movements (Blessing et al. 2010). Chromosomes were stained with 5% Giemsa in phosphate buffer (pH 6.8) for karyotyping, taking into account that metacentric (m), submetacentric (sm) and subtelocentric (st) are biarmed and acrocentric (a) chromosomes are one-armed (Levan et al. 1964).

C-banding (Sumner 1972) was performed to detect heterochromatic regions while silver nitrate staining was carried out to reveal active nucleolus organizer regions (Ag-NORs) as proposed by Howell and Black (1980). Chromosomes were stained with base-specific fluorochromes to detect GC-rich and AT-rich regions by using chromomycin A_3_ (CMA_3_) and 4’6-diamidino-2-phenylindole (DAPI), respectively, with addition of Distamycin A (DA) as counterstain (Schmid 1980).

Fluorescence *in situ* hybridization using simultaneous 18S and 5S rDNA probes (double-FISH) followed the procedure reported by Pinkel et al. (1986) under high stringency conditions (77%). The 18S rDNA probe from *Prochilodus argenteus* Spix & Agassiz, 1829 (Hatanaka and Galetti 2004) was labeled with 16-dUTP–biotin (Roche) while the 5S rDNA probe obtained from *Leporinus elongatus* Valenciennes, 1850 (Martins and Galetti 1999) was labeled with digoxigenin-11-dUTP by nick translation.

The hybridization mix comprised 1 µg of each DNA probe, 10 mg/ml dextran sulfate, 2xSSC, and 50% formamide to a final volume of 30 µl. The mix was dropped onto previously denaturated chromosomes in 70% formamide/2xSSC. Hybridization was carried out overnight at 37 °C in a dark moist chamber. The hybridization signal of 18S and 5S rDNA probes was detected with fluorescein isothiocyanate-avidin conjugate (Sigma-Aldrich®) and anti-digoxigenin-Rhodamine conjugate (Roche®), respectively. Chromosomes were counterstained using DAPI (0.2 mg/mL) in Vectashield Mounting Medium (Vector®) and slides were stored in a dark chamber up to analyses.

All metaphases were photographed by using an Olympus BX-51 epifluorescence microscope equipped with digital camera. Chromosomal images were digitalized in the software IMAGE-PRO PLUS® 6.2.

## Results

**Figure 2.**
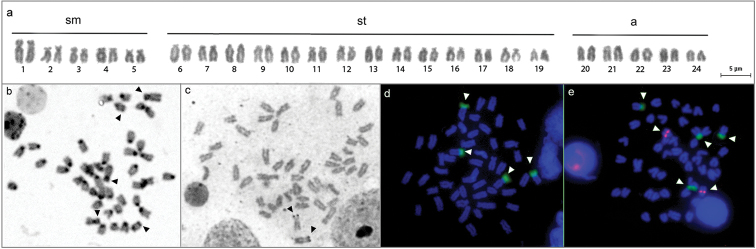
Giemsa-stained karyotype (**a**) and metaphases of *Cichlasoma sanctifranciscense* after C-banding highlighting some non-pericentromeric heterochromatic segments (**b**), silver nitrate staining with single Ag-NORs (**c**), base-specific fluorochrome staining with four CMA_3_^+^ sites (**d**) and FISH with 18S rDNA (green) and 5S rDNA (pink) probes (**e**), as indicated by arrows.

All specimens of *Cichlasoma sanctifranciscense* shared similar chromosomal features independently of collection sites or hydrographic basins. Both males and females presented a modal diploid number of 2n=48 with a karyotype formula of 10sm+28st+10a and a fundamental arm number of FN=86 (Fig. 2a). Heterochromatin segments were invariably more conspicuous in the pericentromeric region, even though some terminal C-bands could be observed at short and long arms of a few chromosomal pairs (Fig. 2b). Active NORs, as revealed by silver nitrate staining, were observed on short arms of a submetacentric pair (equivalent to pair 1), indicating a single active NOR system (Fig. 2c).

On the other hand, GC-rich regions, i.e. repetitive sequences positively stained by CMA_3_ and negatively stained by DAPI, were identified at terminal regions on short arms of four chromosomes, including the sm pair bearing active NORs and a st pair (Fig. 2d). Unfortunately, this additional st pair could not be precisely defined because of the subtle size differences among chromosomes, but it was putatively equivalent to pair 6. Similarly to CMA_3_ staining, double-FISH revealed two pairs bearing 18S rDNA clusters in *Cichlasoma sanctifranciscense*, coincident with Ag-NORs in the first sm pair and another on short arms of a st pair (probably the 6^th^ pair), thereby characterizing a multiple NOR system in this species (Fig. 2e).

Furthermore, the simultaneous hybridization of 18S and 5S rDNA probes showed that 5S rRNA genes are non-syntenic to NORs, occupying the interstitial region of two large acrocentric chromosomes (probably pair 20) (Fig. 2e).

## Discussion

The modal number (2n=48) in *Cichlasoma sanctifranciscense* follows the plesiomorphic pattern reported in the majority of studied cichlids (Feldberg et al. 2003), suggesting a conservative chromosomal evolution in relation to diploid values (Affonso and Galetti 2005). On the other hand, the high number of biarmed chromosomes in spite of the predominance of 2n=48 in Cichlasomatini (Suppl. material 1: Table S1) reveals that pericentric inversions have played a major role in the cytogenetic diversification of this tribe. Indeed, Cichlasomatini is characterized by a remarkable variation in arm number, even though chromosomal condensation and author’s criteria on chromosome morphology might lead to some bias in karyotype formulae differences (Bitencourt et al. 2012). Moreover, some representatives in Cichlasomatini diverge from the general trend observed in most cichlids and Perciformes in general, since some cases of centric fusions or fissions have been described, determining diploid values lower or higher than 48, respectively (Roncati et al. 2007, Schneider et al. 2012, Hodaňová et al. 2014 among others).

Another chromosomal peculiarity of *Cichlasoma sanctifranciscense* refers to 18S rDNA cistrons, since multiple sites were observed by FISH (Fig. 2e). With a few exceptions, cichlids are characterized by a single NOR-bearing pair, usually the largest one (Feldberg et al. 2003).

It should be pointed out that most cytogenetic reports in cichlids describe only silver-stained NORs (e.g. Molina et al. 2014), thereby hindering the actual number of ribosomal cistrons when inactive rDNA regions are present. On the other hand, the hybridization *in situ* with ribosomal probes allows detection of different patterns of NOR distribution in some cichlids (Poletto et al. 2010, Schneider et al. 2012). Similarly, the number of 18S rDNA in *Cichlasoma sanctifranciscense* after FISH was higher than that observed by conventional silver nitrate staining (Ag-NORs) (Fig. 2). Multiple NORs have also been detected in other Cichlasomatini like *Cichlasoma amazonarum* Kullander, 1983 (Salgado et al. 1995) as well as *Aequidens* C. H. Eigenmann & W. L. Bray, 1894 and *Laetacara* Heckel, 1840 (Poletto et al. 2010). This unusual 18S rDNA distribution places this tribe as a divergent group within Cichlidae (Gornung 2013) and further studies using, for instance, mapping of retrotransposons interspersed to NORs might elucidate the dispersal mode of ribosomal cistrons.

Furthermore, the CMA_3_^+^/DAPI signals observed in *Cichlasoma sanctifranciscense* were coincident to 18S rDNA sites, reinforcing that NORs in fishes are usually associated with GC-rich heterochromatin (Verma et al. 2011). In the present study, the base-specific fluorochrome staining was more precise than Ag-NOR to detected 18S rRNA genes. This is an atypical situation in fish and raises the question whether the additional NORs on pair 6 (Fig. 2d) correspond to intact ribosomal cistrons or pseudogenes (Affonso and Galetti 2005).

Differently from 18S cistrons, the 5S rDNA seems to be highly conserved in Cichlidae being primarily located at interstitial region of a single chromosomal pair and non-syntenic to NORs (Gross et al. 2010). The same pattern is described for *Cichlasoma sanctifranciscense*, indicating a basal condition for most fish groups (Martins and Galetti 1999). A putative explanation for the uniformity in both number and location of 5S rRNA genes is the lack of association of these cistrons with heterochromatin observed in most species (Poletto et al. 2010), including the species herein analyzed.

In addition to cytogenetic results, this is the first report about the presence of *Cichlasoma sanctifranciscense* in the Contas River and Itapicuru River basins. Initially, this species was described as endemic to the São Francisco River basin but further studies reported populations of this species in other basins such as Parnaíba, Capivara (Kullander 2003), Tocantins (Lima and Caires 2011) and Recôncavo Sul (Burger et al. 2011). The natural occurrence of *Cichlasoma sanctifranciscense* in other coastal and isolated drainages such as those herein sampled might reflect several headwater captures during evolutionary history of each basin. This process can be caused by vicariant events such as geophysical uplift, landslide followed by isolation of streams or watershed erosion (Albert and Crampton 2010). Moreover, endemic tropical fish species to large riverine systems such as São Francisco River basin should be interpreted with caution since the ichthyofauna composition of smaller and isolated basins in northeastern Brazil remain poorly studied.

In conclusion, we provide the first cytogenetic report in *Cichlasoma sanctifranciscense*, adding new data about the trends of chromosomal evolution of Cichlidae. The present results are also useful to cytotaxonomic studies since peculiar species-specific cytogenetic features combined with absence of interpopulation differences are described. Based on the available karyotypic data in Cichlasomatini, which includes structural and numerical rearrangements as well as dynamic organization of ribosomal cistrons, this tribe can be characterized by high chromosomal evolutionary rates. This evidence, as corroborated by recent reports (Hodaňová et al. 2014) challenges the traditional view that cichlids fish are cytogenetically conserved. Finally, further investigations should be carried out to determine the reason(s) why additional 18S rDNA clusters remain silenced in *Cichlasoma sanctifranciscense*.

## Supplementary materials

Supplementary material 1 Table S1. Cytogenetic data in Cichlasomatini Data type: **cytogenetic data** This dataset is made available under the Open Database License (http://opendatacommons.org/licenses/odbl/1.0/). The Open Database License (ODbL) is a license agreement intended to allow users to freely share, modify, and use this Dataset while maintaining this same freedom for others, provided that the original source and author(s) are credited.Leandro A. Argôlo, Paulo Roberto Antunes de Mello Affonso
