# Supplementary material for: First cytogenetic report in Cichlasoma sanctifranciscense Kullander, 1983 (Perciformes, Cichlidae) from northeastern Brazil with inferences on chromosomal evolution of Cichlasomatini
Source: Comp Cytogenet. 2015 Oct 7;9(4):671–81. doi: 10.3897/CompCytogen.v9i4.5562 (PMC4698579; doi:10.3897/CompCytogen.v9i4.5562)
Supplement: Supplementary material 1 — Table S1. Cytogenetic data in Cichlasomatini [file CompCytogen-9-671-s001.docx]

**Table 1** Cytogenetic data in Cichlasomatini, including diploid number (2n); karyotype formula; fundamental arm number (FN); number of NOR-bearing chromosomes (NORs); heterochromatin distribution (C-bands); GC-rich sites (CMA_3_^+^); and number of 18S and 5S rDNA signals by FISH. *Scientific names refer to current taxonomic identification according to Kullander (2003) and “p” and “c” refer to pericentrometric and centromeric C-bands, respectively.

| **Species*** | **2n** | **Karyotype** | **FN** | **NORs** | **C-bands** | **CMA_3_^+^** | **18S** | **5S** | **References** |
| --- | --- | --- | --- | --- | --- | --- | --- | --- | --- |
| *Acaronia nassa* Heckel, 1840 | 50 | 50st/a | 50 | 2 | p |  |  |  | Santos 2006 |
| *A. nassa* | 50 | 4m/sm+46st/a | 54 | 2 | p |  | 2 | 2 | Schneider et al. 2012 |
| *Acaronia* sp. Heckel, 1840 | 48 | 48st/a | 48 | 2 |  |  |  |  | Salgado et al. 1995 |
| *Aequidens metae* Eigenmann, 1922 | 48 | 6m/sm+42st/a | 54 |  |  |  |  |  | Thompson 1979 |
| *A. metae* | 48 | 6m/sm+42st/a | 54 |  |  |  |  |  | Marescalchi 2005 |
| *Aequidens (Andinoacara) biseriatus* Regan, 1913 | 48 |  |  |  |  |  |  |  | Krajáková et al. 2010 |
| *Aequidens plagiozonatus* Kullander, 1984 | 48 | 12m/sm+36st/a | 60 |  |  |  |  |  | Poletto et al. 2010 |
| *Aequidens (Andinoacara) pulcher* Gill, 1858 | 48 |  | 82 |  |  |  |  |  | Scheel 1973 |
| *A. (Andinoacara) pulcher* | 48 | 4m/sm+44st/a | 52 |  |  |  |  |  | Marescalchi 2005 |
| *A. (Andinoacara) pulcher* | 48 |  |  |  |  |  |  |  | Krajáková et al. 2010 |
| *Aequidens (Andinoacara) rivulatus* Günther, 1860 | 48 |  |  |  |  |  |  |  | Krajáková et al. 2010 |
| *A. (Andinoacara) rivulatus* | 48 | 8m/sm+40st/a | 56 |  |  |  |  |  | Marescalchi 2005 |
| *Aequidens tetramerus* Heckel, 1840 | 48 |  |  |  |  |  |  |  | Marescalchi 2005 |
| *A. tetramerus* | 48 | 12m/sm+36st/a | 60 | 2 |  |  |  |  | Poletto et al. 2010 |
| *Aequidens tubicen* Kullander & Ferreira, 1991 | 48 |  |  |  |  |  |  |  | Krajáková et al. 2010 |
| *Bujurquina peregrinabunda* Kullander, 1986 | 50 | 10m/sm+40st/a | 60 | 2 | p |  |  |  | Santos 2006 |
| *B. peregrinabunda* | 50 | 20m/sm+30st/a | 70 | 2 | p |  | 2 | 2 | Schneider et al. 2012 |
| *Bujurquina vittata* Heckel, 1840 | 44 | 22m/sm+8st/a+1-4 Bs | 66 | 2 | p |  |  |  | Roncati et al. 2007 |
| *B. vittata* | 44 | 26m/sm+19st/a | 71 |  |  |  |  |  | Thompson 1979 |
| *B. vittata* | 44 | 26m/sm+19st/a | 71 |  |  |  |  |  | Marescalchi 2005 |
| *Cichlasoma amazonarum* Kullander, 1983 | 48 | 2m/sm+46st/a | 50 | 3 |  |  |  |  | Salgado et al. 1995 |
| *C. amazonarum* | 48 |  |  |  |  |  |  |  | Krajáková et al. 2010 |
| *Cichlasoma beani* Jordan, 1889 | 48 | 6m/sm+42st/a | 54 |  |  |  |  |  | Thompson 1979 |
| *Cichlasoma bimaculatum* Linnaeus, 1758 | 48 | 6m/sm+42st/a | 54 |  |  |  |  |  | Thompson 1979 |
| *Cichlasoma dimerus* Heckel, 1840 | 48 |  |  |  |  |  |  |  | Roncati et al. 2007 |
| *Cichlasoma (Australoheros) facetus* Jenyns, 1842 | 48 | 22m/sm+26st/a | 70 | 2 | p | 2 | 2 |  | Perazzo et al. 2011 |
| *C. (Australoheros) facetus* | 48 | 8m/sm+40st/a | 56 |  |  |  |  |  | Oyhenart-Perera et al. 1975 |
| *C. (Australoheros) facetus* | 48 | 10m/sm+38st/a | 58 | 2 |  |  |  |  | Feldberg and Bertollo 1985a,b |
| *C. (Australoheros) facetus* | 48 | 10m/sm+38st/a | 58 | 2 |  |  |  |  | Feldberg and Bertollo 1985a,b |
| *C. (Australoheros) facetus* | 48 | 10m/sm+38st/a | 58 | 2 |  |  |  |  | Vicari et al. 2006 |
| *C. (Australoheros) facetus* | 48 | 6m/sm+42st/a | 54 |  |  |  |  |  | Poletto et al. 2010 |
| *Cichlasoma (Amatitlania) nigrofasciata* Günther, 1867 | 48 | 8m/sm+40st/a | 56 |  |  |  |  |  | Poletto et al. 2010 |
| *Cichlasoma (Rocio) octofasciata* Regan, 1903 | 48 |  | 96 |  |  |  |  |  | Zahner 1977 |
| *C. (Rocio) octofasciata* | 48 | 6m/sm+42st/a | 54 |  |  |  |  |  | Thompson 1979 |
| *Cichlasoma orientale* Kullander, 1983 | 48 | 6sm+10st+32a | 54 | 2 | c/p | 2 |  |  | Molina et al. 2014 |
| *Cichlasoma paranaense* Kullander, 1983 | 48 | 20m/sm+28st/a | 68 | 2 |  |  |  |  | Martins et al. 1995 |
| *C. paranaense* | 48 | 6m/sm+42st/a | 54 |  |  |  |  |  | Poletto et al. 2010 |
| *Cichlasoma portalegrense* Hensel, 1870 | 48 |  | 82 |  |  |  |  |  | Scheel 1973 |
| *Cichlasoma salvini* Günther, 1862 | 52 |  | 104 |  |  |  |  |  | Zahner 1977 |
| *C. salvini* | 52 | 28m/sm+24st/a | 80 |  |  |  |  |  | Thompson 1979 |
| *Cichlasoma trimaculatum* Günther, 1867 | 48 | 6m/sm+42st/a | 54 |  |  |  |  |  | Thompson 1979 |
| *Cichlasoma (Parachromis) dovii* Günther, 1864 | 48 | 8m/sm+40st/a | 56 |  |  |  |  |  | Thompson 1979 |
| *C. (Parachromis) dovii* | 48 | 8m/sm+40st/a | 56 |  |  |  |  |  | Salas and Boza 1991 |
| *Parachromis (Cichlasoma) friedrichsthalii* Heckel, 1840 | 48 | 6m/sm+42st/a | 54 |  |  |  |  |  | Salas and Boza 1991 |
| *Cichlasoma* *istlanum* Jordan & Snyder, 1899 | 48 | 8m/sm+40st/a | 56 |  |  |  |  |  | Uribe-Alcocer et al. 1999 |
| *Nandopsis* (*Cichlasoma*) *tetracanthus* Valenciennes, 1831 | 48 | 6m/sm+28st+14a | 82 |  |  |  |  |  | Ráb et al. 1983 |
| *Cleithracara maronii* Steindachner, 1881 | 48 |  | 82 |  |  |  |  |  | Scheel 1973 |
| *C. maronii* | 50 |  | 100 |  |  |  |  |  | Zahner 1977 |
| *C. maronii* | 50 | 12m/sm+38st/a | 62 |  |  |  |  |  | Marescalchi 2005 |
| *C. maronii* | 50 | 14m/sm+36st/a | 64 |  |  | 2 |  |  | Hodaňová et al. 2014 |
| *Nannacara (Ivanacara) adoketa* Kullander & Prada-Pedreros, 1993 | 48 | 16m/sm+32st/a | 64 |  |  | 2 |  |  | Hodaňová et al. 2014 |
| *Krobia* sp. “Xingu” Kullander & Nijssen, 1989 | 48 | 12m/sm+36st/a | 60 |  |  |  |  |  | Krajáková et al. 2010 |
| *Laetacara araguaiae* Ottoni & Costa, 2009 | 44 | 4m/sm+40st/a | 48 | 2 | p | 2 |  |  | Valente et al. 2012 |
| *Laetacara curviceps* Ahl, 1923 | 38 |  |  | 2 |  |  |  |  | Scheel 1973 |
| *Laetacara dorsigera* Heckel, 1840 | 43 | 5m/sm+38st/a | 48 | 2 | c |  |  |  | Martins-Santos et al. 2005 |
| *L. dorsigera* | 44 | 4m/sm+40st/a | 48 | 2 | c |  |  |  | Martins-Santos et al. 2005 |
| *L. dorsigera* | 45 | 3m/sm+42st/a | 48 | 2 | c |  |  |  | Martins-Santos et al. 2005 |
| *L. dorsigera* | 46 | 2m/sm+44st/a | 48 | 2 | c |  |  |  | Martins-Santos et al. 2005 |
| *L. dorsigera* | 44 | 22m/sm+22st/a | 66 | 2 |  |  |  |  | Marescalchi 2005 |
| *L. dorsigera* | 44 | 4m/sm+40st/a | 48 | 2 |  |  |  |  | Poletto et al.2010 |
| *Nannacara anomala* Regan, 1905 | 48 |  |  |  |  |  |  |  | Post 1965 |
| *N. anomala* | 44 |  |  |  |  |  |  |  | Scheel 1973 |
| *N. anomala* | 44 | 18m/sm+26st/a | 62 |  |  |  |  |  | Thompson 1979 |
| *N. anomala* | 44 | 18m/sm+26st/a | 62 |  |  | 2 |  |  | Hodaňová et al. 2014 |
| *Nannacara aureocephalus* Allgayer, 1983 | 44 | 18m/sm+26st/a | 62 |  |  | 6 |  |  | Hodaňová et al. 2014 |
| *Nannacara taenia* Regan, 1912 | 44 | 16m/sm+28st/a | 60 |  |  | 2 |  |  | Hodaňová et al. 2014 |
| *Tahuantinsuyoa macantzatza* Kullander, 1986 | 30 |  |  |  |  |  |  |  | Krajáková et al. 2010 |
